# Exploring the Antimicrobial and Antitumor Potentials of *Streptomyces* sp. AGM12-1 Isolated from Egyptian Soil

**DOI:** 10.3389/fmicb.2017.00438

**Published:** 2017-03-13

**Authors:** Maged S. Ahmad, Ahmed O. El-Gendy, Rasha R. Ahmed, Hossam M. Hassan, Hussein M. El-Kabbany, Ahmed G. Merdash

**Affiliations:** ^1^Botany and Microbiology Department, Faculty of Science, Beni-Suef UniversityBeni-Suef, Egypt; ^2^Microbiology and Immunology Department, Faculty of Pharmacy, Beni-Suef UniversityBeni-Suef, Egypt; ^3^Zoology Department, Faculty of Science, Beni-Suef UniversityBeni-Suef, Egypt; ^4^Pharmacognosy Department, Faculty of Pharmacy, Beni-Suef UniversityBeni-Suef, Egypt; ^5^Health Research Department, The National Center for Radiation Research and Technology, Atomic Energy AuthorityBeni-Suef, Egypt

**Keywords:** actinomycetes, antimicrobial, antitumor, diketopiperazine, *Streptomyces* sp., *Streptomyces vinaceusdrappus*

## Abstract

The occurrence of extensive antibiotics resistant bacteria increased the demands for mining out new sources of antimicrobial agents. Actinomycetes, especially *Streptomyces* sp. have grasped considerable attention worldwide due to production of many useful bioactive metabolites. In the present study, a total of 52 actinomycetes were isolated from agricultural soil samples in Beni-Suef, Egypt. All isolates were characterized based on colony morphology, mycelium coloration, and pigment diffusion. They were screened for their capabilities to show antimicrobial activities against different indicator microorganisms, and only 20 isolates have shown significant antimicrobial activities against at least one of the tested indicator microorganisms. The isolate AGM12-1 was active against all tested microorganisms and showed a marked antitumor activity with IC_50_ 3.3 and 1.1 μg/ml against HCT-116 and HepG-2 cell lines respectively. It was genotypically characterized as *Streptomyces* sp. with the presence of PKS Π biosynthetic gene cluster. Mannitol, ammonium sulfate, pH 7, 2% inoculum size and incubation for 11 days at 30°C were the optimum conditions that used to maximize the production and hence allowed purification of one active antimicrobial compound to homogeneity using high performance liquid chromatography with a molecular mass of m/z 488.05. Nuclear magnetic resonance structural elucidation showed that this compound was a diketopiperazine derivative.

## Introduction

Actinomycetes are Gram-positive filamentous bacteria with fungal morphology. They are characterized by a complicated life cycle belonging to the phylum *Actinobacteria* (Dilip et al., 2013). They are widely distributed in terrestrial ecosystems, especially in soil, where they play a pivotal role in recycling of industrial wastes and biomaterials by decomposing complex polymeric structures in dead plants, animals, and fungal materials (Goodfellow and Williams, 1983). Actinobacteria, especially *Streptomyces* sp. are known as noble factories for the production of many biologically active compounds that are useful as antibacterials, antifungals, antivirals, antithrombotics, immunomodifiers, anti-tumor drugs, and enzyme inhibitors in many fields especially in medicine (Sacramento et al., 2004; Atta, 2009; Fukuchi et al., 2009; Olano et al., 2009; Ser et al., 2015). Due to the emergence of multi-resistant microorganisms to almost all available antibiotics, many researchers are focused now on discovering novel antimicrobials from many natural resources such as those produced by actinomycetes especially those isolated from many undiscoverable or poorly explored environments (Undabarrena et al., 2016). These antimicrobials are produced by many metabolic pathways, mainly organized by polyketide synthases (PKS) and non-ribosomal peptide synthetases (NRPS) (Undabarrena et al., 2016). Incidence and presence of these biosynthetic genes in actinobacteria are obviously high (Donadio et al., 2007).

Egyptian soil reservoir is considered as a poorly investigated source for actinobacteria, and very few reports were published (Hozzein and Goodfellow, 2007; Awad et al., 2009; Abd-Alla et al., 2013; Rifaat et al., 2013). In these perspectives, the present study aimed to isolate and characterize different actinomycetes from soil niche in Beni-Suef governorate, Egypt. Actinomycetes were screened for their capabilities to produce antimicrobial and antitumor active metabolites. The bioactive compound from the most potent isolate was further purified and characterized.

## Materials and Methods

### Collection of Samples and Isolation of Actinomycetes

A number of 13 agricultural soil samples were collected aseptically from four different sites located in Egypt (Beni-Suef City, Beba City, Ehnasia City, and El-Fayum roads) from the upper 15 cm layer of soil using sterile plastic bags and transported to the laboratory for further isolation steps. Isolation of actinomycetes was managed using soil dilution plate technique (Williams et al., 1983) on starch nitrate agar (SNA) and tryptone soya agar (TSA) supplemented with Rifampicin (10 μg/ml) and Nystatin (50 μg/ml) to inhibit any bacterial or fungal contaminants. Briefly, 1 g of each soil sample was diluted with 9 ml of 0.9% saline, homogenized and then a serial dilution up to 10^-4^ was carried out. A 100 μl from 10^-2^, 10^-3^, and 10^-4^ dilutions were spread on SNA and TSA, and incubated at 30°C for 7 days. Suspected colonies of actinomycetes were characterized morphologically (Aghamirian and Ghiasian, 2009; Reddy et al., 2011), re-purified using streak plate method, and then stored in 40% glycerol at -80°C.

### Screening of Actinomycetes for their Antimicrobial Activities

Antimicrobial activities of pure isolates were determined by agar diffusion method (Williams and Davies, 1965) and double layer agar method (modified spot on lawn technique) (Thakur et al., 2007; Dundar et al., 2015), against different indicator microorganisms; *Escherichia coli* (ATCC 8739), *Staphylococcus aureus* (ATCC 6538), *Bacillus subtilis* (clinical sample), *Candida albicans* (clinical sample), and *Sarcina lutea* (environmental sample). In double layer agar method, all pure isolates were spot inoculated on SNA and incubated at 30°C for 5 days and then 5 ml of molten TSA seeded with 100 μl overnight culture of indicator microorganisms were poured on spotted plates and incubated at 37°C for 24 h. Antimicrobial activities represented in zones of inhibitions were examined.

### Fermentation and Extraction of Secondary Metabolites and Total Proteins

The most potent actinomycetes, showing significant antimicrobial activities, were undergoing fermentation. Briefly, actinomycetes were sub-cultured in tryptone soya broth (TSB) for 5 days and 2% of starting inoculum was used to inoculate 1 L of International Streptomyces Project 4 (ISP4) broth in a 2 L Erlenmeyer flask and then incubated on rotary shaker incubator (200 RPM) at 30°C for 7 days. The cell-free supernatant from each flask was collected after centrifugation at 13,000 *g* for 20 min and divided into two portions; one used to extract total secondary metabolites, and the other used to extract total proteins. For extraction of total metabolites, a 1:1 v/v ethyl acetate was added to the cell-free culture supernatant and shacked vigorously for 1 h. The organic phase was separated and evaporated to dryness using a rotary evaporator (Romankova et al., 1971; Selvameenal et al., 2009). Total extract residues were weighed and dissolved in 5 ml ethyl acetate and kept in a refrigerator at 4°C. For extraction of total proteins, an ammonium sulfate was added to the cell-free culture supernatant in a concentration of 40%. Protein pellets were collected by centrifugation at 14,000 *g* for 30 min at 4°C and then dissolved in 5 ml distilled water, and kept in a refrigerator at 4°C.

### *In vitro* Anti-tumor Cytotoxicity

Both ethyl acetate extracts and total proteins of the most potent actinomyces isolates with significant antimicrobial activities were evaluated for their cytotoxicity using tissue culture technique. HepG2 (hepatocellular carcinoma cell line) and HCT 116 (human colon carcinoma) were obtained from the Pharmacology Unit, Cancer Biology Department, National Cancer Institute, Cairo University, Egypt. Cells were maintained in DMEM medium with 10% fetal calf serum, sodium pyruvate, 100 U/ml penicillin and 100 mg/ml streptomycin at 37°C and 5% CO_2_ till the cytotoxicity bioassay was carried out. The potential cytotoxicity of four samples was tested using the method of Skehan et al. (1990). Briefly, 100 cells/well were plated onto 96-well dishes overnight before the treatment with the tested compounds to allow the attachment of cells to the wall of the plate. Different concentrations of each tested compound (0, 6.25, 12.5, 25, 50, 100 μg/ml) were added to the cell monolayer and triple wells were used for each individual dose. Monolayer cells were incubated with the tested agent(s) for 48 h at 37°C and 5% CO_2_. At the end of the incubation period, the cells were fixed and stained with sulforhodamine B dissolved in acetic acid. Unbound stain was removed by washing four times with 1% acetic acid and the protein-bound dye was extracted with Tris-EDTA buffer. The absorbance was measured in an ELISA reader. The relation between surviving fraction and compound concentration was plotted to get the survival curve of each tumor cell line and the IC_50_. The concentration of an agent that causes a 50% growth inhibition, for each tested agent using each cell line was obtained from the survival curve (Skehan et al., 1990).

### Phenotypic Characterization of AGM12-1 Isolate

The physiological, biochemical, and cultural characteristics of the talented isolate AGM12-1 which showed broad antimicrobial and cytotoxic activities were examined in detail. The growth capability, pigment production, and color of both aerial and substrate mycelium was determined using different growth media; ISP-3, ISP-4, ISP-5, Czapek Dox, Sato, nutrient agar and SNA. The color was determined visually by making a comparison with chips from the ISCC-NBS centroid color charts (Williams et al., 1983). The types of spore-bearing hyphae and spores chain morphology were determined using direct microscopical examination and the shape of the spore surface was observed using scanning electron microscope (SEM). Production of catalase, lecithinase, protease, lipase, pectinase, amylase, hydrogen sulfide, nitrate reductase, urease, gelatinase, and melanin besides screening for utilization of different nitrogen sources (peptone, protease peptone, potassium nitrate, yeast extract, ammonium sulfate), different carbon sources (starch, glucose, sucrose, fructose, mannitol) and ability to grow at a wide range of pH from 5 to 11 were carried out according to Williams et al. (1983).

### Genomic DNA Extraction and Purification

Genomic DNA extraction was done according to Sinha et al. (2004) and Aly et al. (2016) with some modifications. Briefly, a 1.5 ml of culture was centrifuged for 10 min at 3,000 *g*, the supernatant was discarded and the pellets were resuspended in 200 μl spheroblast buffer (10% sucrose, 25 mM Tris pH 8.4, 25 mM EDTA pH 8.0, 2 mg/mL lysozyme and 0.4 mg/ml RNase A), vortexes and incubated at 37°C for 10 min until cell lysis occurred. Then, 50 μl of 5% SDS (lysis buffer 1) and 5 M NaCl (lysis buffer 2) were added, mixed and incubated at 65°C for 5 min. A 100 μl neutralizing buffer (60 ml 5 M Potassium acetate, 11.5 ml glacial acetic acid, and 28.5 ml dH_2_O) was then added and put on ice for 5 min before centrifugation at 18,000 *g* at 4°C for 15 min. The supernatant (approximately 400 μl) was transferred to a new tube, mixed with equal volume of isopropanol, left 5 min at room temperature and centrifuged at 18,000 *g* at room temperature for 15 min to precipitate the DNA. The resulting pellet was washed with 70% ethanol by centrifugation at 18,000 *g* at room temperature for 5 min. The final pellet was air-dried and resuspended in 50 μl 1x TE buffer pH 8 and stored in the refrigerator at 4°C.

### PCR Amplification and Sequencing of 16S rRNA Gene

The primers used for amplification of the 16S rRNA gene were 11F: 5′-TAACACATGCAAGTCGAACG-3′ (Birri et al., 2013; Hong-Thao et al., 2016) and 12R: 5′-AGGGTTGCGCTCGTTG-3′ (Stackebrandt and Charfreitag, 1990; Isik et al., 2014). PCR was carried out in 50 μl reaction volume in sterile 200 μl PCR tube. The PCR reaction mixture consisted of 500 ng genomic DNA, 10 mM dNTPs mixture, 1 μl (20 uM of each primer), 2.5 units of Taq DNA polymerase enzyme and 10 μl 5x reaction buffer. The PCR program included template denaturation at 94°C (3 min), followed by 34 cycles of denaturing at 94°C (30 s), annealing at 56°C (30 s), and extension at 72°C (60 s), and followed by completion of DNA synthesis at 72°C (5 min). Primers were removed from the final PCR product prior to sequencing using QIAquick PCR purification kit (QIAGEN, Germany). The PCR product of interest was detected and purified by agarose gel electrophoresis using 1% (w/v) agarose gels with reference to 1 kbp DNA ladder. DNA was sequenced using the ABI Prism BigDye terminator sequencing ready reaction kit version 3.1 and analyzed with the ABI Prism 3100 generic analyzer.

### Sequence Manipulation and Phylogenetic Analysis

The BLAST facility^1^ was employed in order to assess the degree of DNA similarity. Multiple sequence alignment and molecular phylogeny were evaluated using MEGA7 software (Tamura et al., 2007).

### PCR Screening for Antibiotic Biosynthetic Gene Clusters

The genomic DNA of AGM12-1 isolate was screened for the presence of the biosynthetic genes involved in the production of type I polyketide synthase (PKS I), type II polyketide synthase (PKS II), NRPS and glycopeptide antibiotics. This was achieved by PCR amplification of these genes using the following primers; PKS/K1 F: 5′-TSAAGTCSAACATCCGBCA-3′ and PKS/M6 R: 5′-CGCAGGTTSCSGTACCAGTA-3′ to amplify the PKS I gene with expected product size of 1200–1400 bp (Passari et al., 2015), ARO-PKS-F: 5′-GGCAGCGGITTCGGCGGITTCCAG-3′ and ARO-PKS-R: 5′-CGITGTTIACIGCGTAGAACCAGGCG-3′ to amplify the PKS II gene with expected product size of 492–630 bp (Wood et al., 2007), NRPS/A3 F: 5′-GCSTACSYSATSTACACSTCSGG-3′ and NRPS/A7 R: 5′-SASGTCVCCSGTSGCGTAS-3′ to amplify the NRPS gene with expected product size of 700 bp (Passari et al., 2015), and finally oxyB F: 5′-CTGGTCGGCAACCTGATGGAC-3′ and oxyB R: 5′-CAGGTACCGGATCAGCTCGTC-3′ to amplify the glycopeptide antibiotic gene with expected product size of 696 bp (Wood et al., 2007). The PCR program included template denaturation at 95°C (5 min), followed by 40 cycles of denaturing at 95°C (30 s), annealing for PKS I, PKS II, NRPS and glycopeptide primers at 55, 64, 59, and 60°C, respectively (60 s), extension at 72°C (2 min), and followed by completion of DNA synthesis at 72°C (10 min) (Baker et al., 2003).

### Optimization of Antimicrobial Production

In order to maximize the production of secondary metabolites by the isolate AGM12-1, the effect of different carbon sources; fructose, glucose, mannitol, starch, and sucrose (20 g/l) in basal nitrate salt medium were studied (Selvin et al., 2009). The effect of different nitrogen sources; peptone, yeast extract, ammonium sulfate, protease peptone, and KNO_3_ (2 g/l) in the basal starch salt medium were also studied. The most effective carbon and nitrogen sources were further used in different concentrations at (1, 1.5, 2, 2.5, 3 g/100 ml) and (0.1, 0.15, 0.2, 0.25, 0.3 g/100 ml) respectively. The effects of cultural conditions like different incubation time (2–14 days), different starting pH (5, 6, 7, 8, 9, 10, and 11), and different starting inocula (0.01, 0.1, 2, 5, 10, and 15%) were also examined (Kadiri and Yarla, 2016). The antimicrobial activity assay using cup technique against sensitive indicator *Sarcina lutea* was managed after each experiment, and zones of inhibitions were measured after incubation of plates at 37°C for 24 h.

### Fermentation Using Optimized Conditions and Extraction of the Antimicrobial Compound

To extract putative antimicrobial compound, a 20 ml of 5 days sub-cultured broth was inoculated in 2 L Erlenmeyer flasks containing 1 L of liquid mannitol – ammonium sulfate medium (tow flasks). These flasks were incubated in a rotary shaker (160 RPM) at 30°C for 11 days. A 2-L total volume was filtered through Whatman No. 1 filter. After filtration, the total culture filtrate was extracted with ethyl acetate in a ratio of (1:1 v/v) and shaken vigorously in a separating funnel. Then, the organic layer was collected and the solvent extracts were concentrated to dryness using rotary evaporator and tested for their antimicrobial activity against various indicator microorganisms.

### Purification by HPLC

The total ethyl acetate extract of AGM12-1 was concentrated and chromatographed via high performance liquid chromatography (HPLC) (Dionex Ultimate 3000 model HPLC system at the faculty of pharmacy, Beni-Suef University) using a Nucleosil C18 column. Elution was carried out using flow-rate 3 ml/min of 10–100% acetonitrile in water with total run time 25 min. A total of 23 fractions, 3 ml each, were collected. Fractions were concentrated, dried, weighed then dissolved in DMSO and tested for their antimicrobial activities by spotting on the lawn of *Sarcina lutea*, and tested for their minimum inhibitory concentrations by broth micro-dilution method against various indicator microorganisms.

### Spectroscopic Characterization

The LC-Mass spectrum in positive and negative ion mode was determined at the faculty of postgraduate studies of advanced science, Beni-Suef University, Egypt and the nuclear magnetic resonance (NMR) spectrum was determined at the faculty of pharmacy, Beni-Suef University, Egypt (El-Hawary et al., 2016).

## Results and Discussion

The emergence of extensive antibiotics resistant bacteria increased the demands for finding out new sources of antimicrobial agent. Actinomycetes, especially *Streptomyces* sp., have grasped considerable attention worldwide due to the production of many useful bioactive metabolites. Isolation of these species from poorly explored habitats could increase the possibility to discover novel microbial products with new types of activities (Ser et al., 2016b; Sharma et al., 2016).

### Isolation of Actinomycetes and Screening for their Antimicrobial Activities

A total of 52 actinomycetes were isolated from agricultural soil samples collected from different locations in Beni-Suef Governorate, Egypt. Soil niches were reported to be rich in many significant actinomycetes (Savic et al., 2007; Tan et al., 2015). All isolates were characterized based on colony morphology, mycelium coloration, and pigment diffusion. The protein and organic extracts of each strain were screened for antimicrobial activity against different indicator microorganisms. The organic extracts of 20 (38.46%) out of 52 actinomyces isolates showed antimicrobial activity against at least one of the tested indicator microorganisms (Supplementary Tables 1a,b). This percentage was not surprising because it was reported many times before that incidence of actinomycetes with antimicrobial activities from the soil niche was relevant high (Thakur et al., 2007; Bizuye et al., 2013). In this study, four isolates were able to exhibit promising broad spectrum activity against all tested indicator microorganisms especially AGM12-1 isolate. So, these talented isolates were undergoing batch fermentation plus extraction of their secondary metabolites for further assessments.

### *In vitro* Anti-tumor Cytotoxicity

**FIGURE 1:**
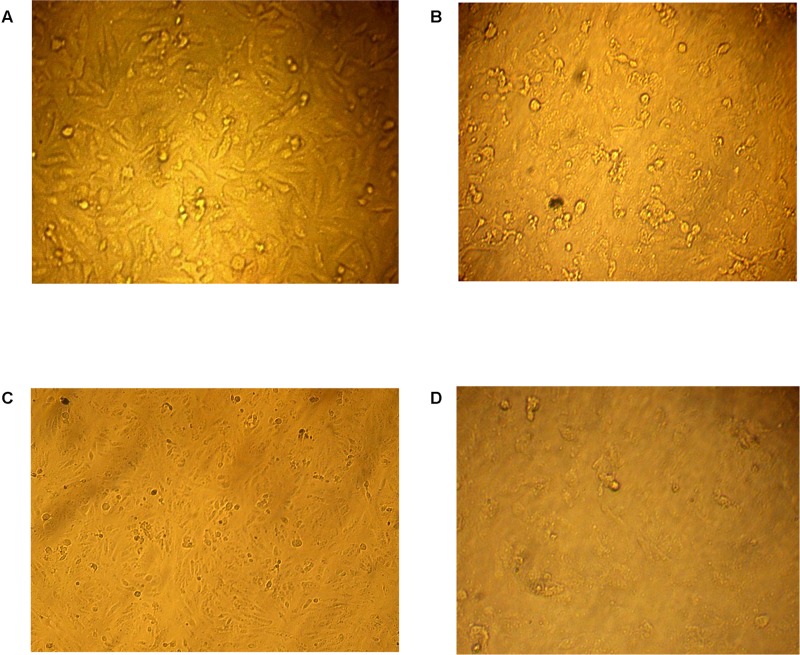
***In vitro* anti-tumor cytotoxicity bioassay.** **(A)** Normal human liver cancer cell line (HepG2) while the effect of three tested concentrations 25, 50, 100 μg/ml of the AGM12-1 extract on the survival percent of HepG-2 are illustrated in **(B–D)**, respectively.

**FIGURE 2:**
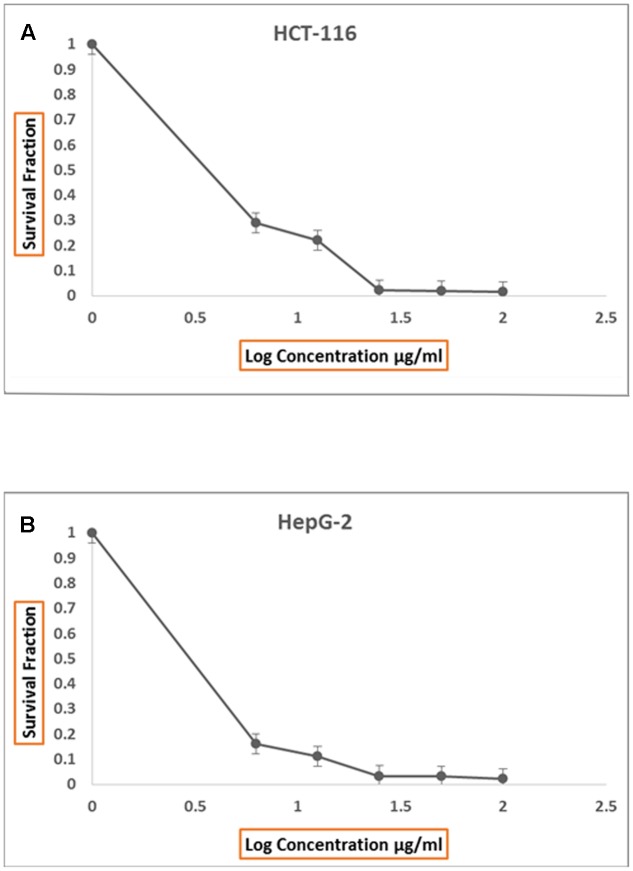
**Anti proliferative effect of total ethyl acetate extract, produced by AGM12-1, on HCT116 cell lines *in vitro*** **(A)** and on HepG2 cell lines *in vitro* **(B)**.

Incessant efforts have been directed at the search for more effective anti-tumors from natural resources which could be settled into new therapeutic drugs (Ser et al., 2016a). In this study, both of protein and organic extracts were screened for anti-tumor cytotoxicity against human liver cancer cell line (HepG2) and human colon carcinoma (HCT116). The organic extracts of 20 isolates showed anti-tumor toxicity against both cell lines. The organic extract of AGM12-1 isolate showed substantial anti-tumor activity (**Figure 1**) where the survival fractions were significantly decreased as the concentration increased (**Figures 2A,B**). The IC_50_ values were reached for all examined extracts using the tested concentrations for both cell lines. The ethyl acetate extract from isolate AGM12-1 exhibited the most potent effect against both cell lines with IC_50_ 3.3 and 1.1 μg/ml against HCT 116 and HepG-2 respectively. Moreover, all tested compounds showed higher cytotoxicity against HepG 2 cell line compared to HCT 116.

### Phenotypic Characterization of AGM12-1 Isolate

The light microscopic observation of AGM12-1 isolate on ISP4 media showed a straight chain section with no fragmentation of the aerial mycelium. Also SEM observation showed a crimpy spore surface with aerial and vegetative hyphae which were well-developed and not fragmented. The other morphological characters, after growing in different growth media, are summarized in **Table 1**. The physiological and biochemical characters denoted in the production of different enzymes, utilization of different nitrogen and carbon sources, and ability to grow at a wide range of pH are illustrated in **Table 2**. Based on these morphological and biochemical characteristics of AGM 12-1, it was presumptively identified as a member of *Streptomyces* sp. according to Williams et al. (1983).

**Table 1:** Morphological characters and growth of *Streptomyces* sp. AGM12-1.

Growth media	Growth	Color of aerial mycelium	Color of substrate mycelium	Diffused pigments	Form of spore chain
Sato medium	Abundant	Light brown	Light yellowish brown	-ve	Rectus
Starch nitrate medium	Good	Medium gray	Light grayish–reddish brown	-ve	Rectus
Czapek dox medium	Good	Light gray	Light yellowish brown	-ve	Rectus
Nutrient agar	Abundant	Medium gray	Dark grayish yellow	-ve	Rectus
ISP 3	Good	Light grayish–yellowish brown	Grayish yellow	-ve	Rectus
ISP 4	Good	Light gray	Light yellowish brown	-ve	Rectus
ISP 5	Good	Grayish–yellowish brown	Pale yellow	-ve	Rectus


**Table 2:** Physiological and biochemical characteristics of AGM12-1 isolate.

Experiment	Reaction by AGM12-1
**The enzymatic activity**	
Catalase activity	**+**
Gelatinase activity	**+++**
H_2_S production	-
Urea decomposition	**++**
Amylase activity	+++
Pectinolytic activity	**+++**
Lethinase activity	-
Lipase activity	-
Nitrate reductase activity	-
**Carbon source utilization**	
Starch utilization	**+++**
Glucose utilization	**++**
Sucrose utilization	**+**
Fructose utilization	**++**
Mannitol utilization	**+++**
**Nitrogen source utilization**	
Peptone utilization	**++**
Protease peptone utilization	**++**
Potassium nitrate utilization	**+**
Yeast extract utilization	**+**
Ammonium sulfate utilization	**+++**
**Growth at different pH**	
pH 5	**+**
pH 6	**+**
pH 7	**+++**
pH 8	**++**
pH 9	**++**
pH 10	**+**
pH 11	**+**
**Melanin production**	-


*-, negative; +, weakly positive; ++, moderately positive; +++, highly positive. Bolded symbols mean: Main experimental category.*

### Genotypic Characterization of AGM12-1 Isolate and Screening for Antibiotic Biosynthetic Gene Clusters

**FIGURE 3:**
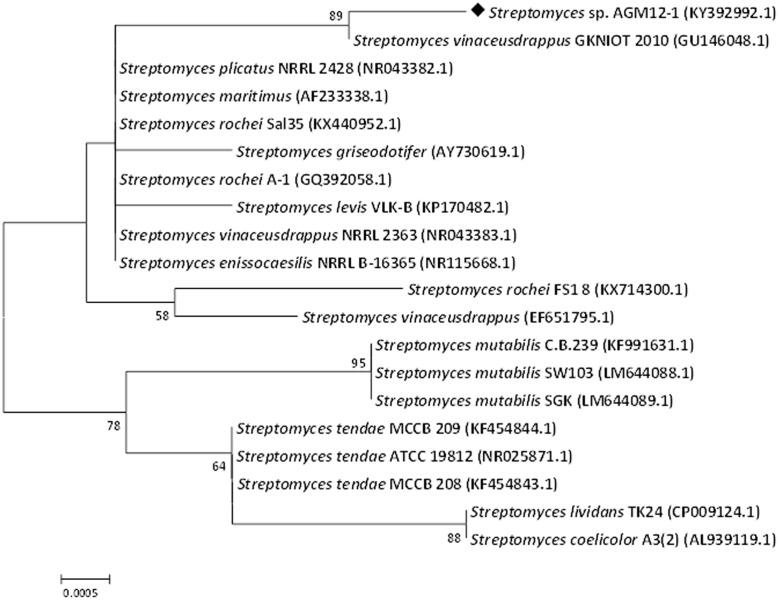
**Phylogenetic tree of AGM12-1 isolate based on partial 16S rRNA gene sequences.** The phylogenetic tree was inferred using the Neighbor-Joining method (Saitou and Nei, 1987). The distances were computed using the Kimura 2-parameter method (Kimura, 1980) and are in the units of the number of base substitutions per site. Numbers at nodes indicate percentages of 1000 bootstrap re-samplings, only values above 50% are shown. The analysis involved 20 nucleotide sequences. Codon positions included were 1st+2nd+3rd+Noncoding. All positions containing gaps and missing data were eliminated. There were a total of 827 positions in the final dataset. Evolutionary analyses were conducted in MEGA7 (Kumar et al., 2016).

The partial 16S rRNA gene sequencing revealed a 99% similarity with *Streptomyces vinaceusdrappus* according to NCBI GenBank. The resulted sequence was aligned to 19 of the closely related *Streptomyces* sp. by retrieving their sequences from the NCBI GenBank database and assembled in MEGA7 software for phylogenetic analysis using the Neighbor-Joining method and the evolutionary distances were computed using the Kimura 2-parameter method. The obtained phylogenetic tree (**Figure 3**) confirmed the similarity of the AGM12-1 isolate to *Streptomyces vinaceusdrappus* with a similarity matrix bootstrap value of 89. The GenBank accession number for the partial 16S rRNA gene sequence of AGM12-1 strain is KY392992.

Screening of *Streptomyces* sp. AGM12-1 for the presence of biosynthetic genes involved in the production of glycopeptide antibiotics (OXY B), NRPS, type I polyketide synthase (PKS I) and type II polyketide synthase (ARO-PKS II) revealed the presence of type II polyketide synthase system which is mostly responsible for the synthesis of aromatic polyketides. In another study (Busti et al., 2006), they reported the presence of antibacterial activated genes NRPSs, type I and type II PKSs in their isolate.

### Optimizing the Production of Antimicrobial Secondary Metabolites

The optimum growth conditions for production of antimicrobial and antitumor agents from *Streptomyces* sp. AGM12-1 were screened and illustrated in **Table 3**. It was found that the maximum productivity was achieved after using mannitol and ammonium sulfate at concentrations of 2.5 and 0.2%, respectively. Other factors like pH 7, starting inoculum 2% and incubation for 11 days at 30°C were found to produce a high yield of antimicrobial and antitumor substance. In other studies (Sujatha et al., 2005), it was reported that the glucose and ammonium nitrate in synthetic media were the optimum carbon and nitrogen sources to obtain a high yield of antibiotic. Also (Kadiri and Yarla, 2016) reported that the arabinose and dextrose were the best carbon sources and L-asparagine was the best nitrogen source in their study. Pandey et al. (2005) tested a number of carbon and nitrogen compounds for their effect on the production of an antibacterial antibiotic by *Streptomyces kanamyceticus* M27. It was found dextrose as the most suitable carbon source while maltose, sucrose, and soluble starch gave moderate yield. (NH_4_)H_2_P0_4_ and yeast extract were adequate nitrogen sources for antibiotic production. It was found that media with alkaline pH gave high antibiotic yield.

**Table 3:** Different growth conditions of *Streptomyces* sp. AGM12-1 and their impacts on antimicrobial agent production measured by inhibition zones against *Sarcina lutea.*

Different growth conditions	Inhibition zone in millimeter
Starch	19
Glucose	15
Sucrose	14
Fructose	18
Mannitol	20
Potassium nitrate	19
Peptone	24
Yeast extract	22
Ammonium sulfate	26
Protease peptone	24
Mannitol 1%	26
Mannitol 1.5%	26
Mannitol 2%	28
Mannitol 2.5%	29
Mannitol 3%	28
Ammonium sulfate 0.1%	25
Ammonium sulfate 0.15%	27
Ammonium sulfate 0.2%	29
Ammonium sulfate 0.25%	26
Ammonium sulfate 0.3%	25
pH 5	14
pH 6	15.5
pH 7	22
pH 8	19
pH 9	17
pH 10	16
pH 11	11
Starting inoculum 0.01%	NA
Starting inoculum 0.1%	NA
Starting inoculum 2%	22
Starting inoculum 5%	15.5
Starting inoculum 10%	11
Starting inoculum 15%	11
Incubation period 2nd day	NA
Incubation period 3rd day	12
Incubation period 4th day	15
Incubation period 5th day	18
Incubation period 6th day	19
Incubation period 7th day	19
Incubation period 8th day	20
Incubation period 9th day	21
Incubation period 10th day	22
Incubation period 11th day	25
Incubation period 12th day	23
Incubation period 13th day	22
Incubation period 14th day	21


### Purification of the Antimicrobial Compound and MIC Determination

**FIGURE 4:**
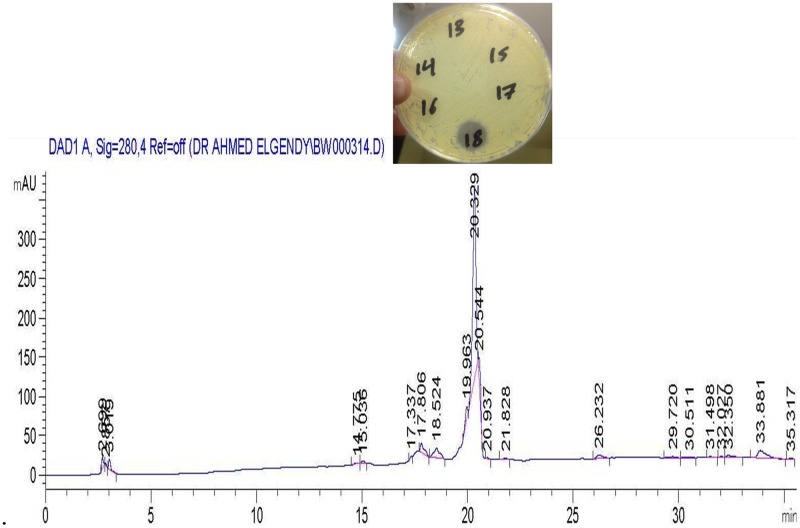
**High performance liquid chromatography (HPLC) chromatograms of a re-purified sample using a Nucleosil C18 column showing the active compound eluted at 20.32 min (fraction tube number 18)**.

The active metabolites were extracted with ethyl acetate at the level of (1:1 v/v) and the separation of the antimicrobial compound was carried out by HPLC in which 23 fractions were collected manually and tested for their antimicrobial activities (Supplementary Figure 1). The active fractions were re-chromatographed till showing one pure compound at 20 min (fraction number 18) as seen in **Figure 4**. MIC values, ranging from 50 to 0.77 μg/ml, were tested against all indicator microorganisms by broth-micro dilution method. Lowest MIC was recorded against *Sarcina lutea* (6.25 μg/ml) while largest MIC was recorded against *Candida albicans* and *Escherichia coli* ATCC 8739 (25 μg/ml). For both *Staphylococcus aureus* ATCC 6538 and *Bacillus subtilis*, the recorded MIC was 12.5 μg/ml.

### Spectroscopic Characteristics

**FIGURE 5:**
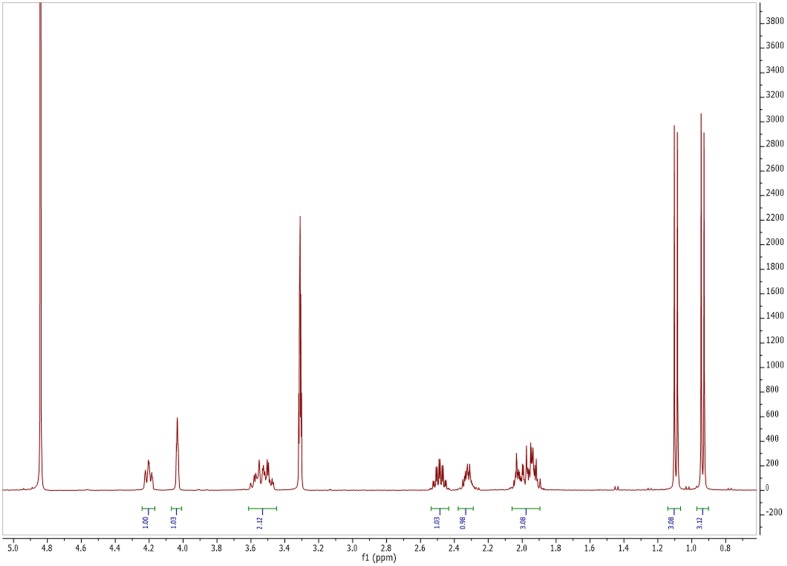
**Nuclear magnetic resonance (NMR) spectrum of antimicrobial and antitumor agent produced by *Streptomyces* sp.** AGM12-1.

**FIGURE 6:**
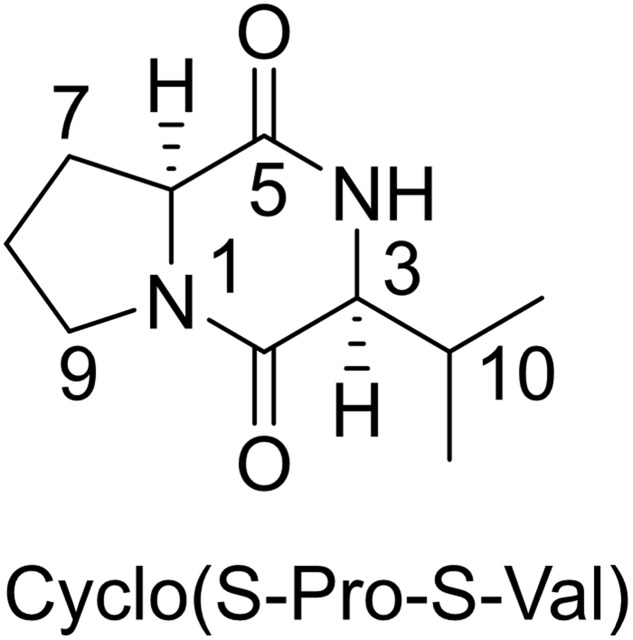
**Chemical structure of the identified compound (a derivative of diketopiperazine) from *Streptomyces* sp. AGM12-1**.

Based on ^1^HNMR spectrum (**Figure 5**), molecular mass (m/z 488.05) and comparing the results with previously published data, the isolated compound was identified as Cyclo (S-Pro-S-Val) (**Figure 6**) (Jayatilake et al., 1996). This compound is related to the diketopiperazine family that has important biological activities as inhibition of plasminogen activator inhibitor-1 (PAI-1) (Einholm et al., 2003) and alteration of cardiovascular and blood-clotting functions (Martins and Carvalho, 2007). They also have activities as antitumor, antiviral, antifungal, antibacterial, and antihyperglycaemic (Martins and Carvalho, 2007).

Diketopeprazine is a huge family with variable bioactivities and about 200 articles had isolated many of these derivatives. For examples, Wang et al. (2013) reported that five new diketopiperazine derivatives were isolated from the marine-derived actinomyces “*Streptomyces* sp. FXJ7.328.” In another study, researchers reported that five diketopiperazines derivatives were isolated from deep-sea bacterium *Streptomyces fungicidicus* with a novel antifoulants activity (Li et al., 2006). Also, *Streptomyces globisporus* 1912, a producer of the antitumor antibiotic landomycin E, forms new low molecular signaling molecule *N*-methyl phenylalanyl-dehydrobutyrine diketopiperazine (Matselyukh et al., 2012).

## Conclusion

Actinomycetes, especially streptomycetes, still an important source for bioactive compounds that are used for treating infections, cancer, and many other diseases. The derivative of diketopiperazine produced by *Streptomyces* sp. AGM12-1, isolated from Beni Suef Governorate, Egypt, demonstrated obvious inhibitory effects against both Gram-positive and Gram-negative bacteria beside an antifungal activity. Also, an anti-tumor toxicity against human liver and colon cell lines; HepG 2 and HCT 116 was reported. To our knowledge, this is the first time to characterize a diketopiperazine derivative as a secondary metabolite recovered from *Streptomyces* sp. in Egypt.

## Author Contributions

MA and AE-G performed the microbiological and molecular biology experiments. RA worked on antitumor activity assay. HH manipulated the NMR data and elucidated the final molecular structure. HE-K and AM put the study design. AE-G drafted the manuscript and all authors revised and approved the final manuscript.

## Conflict of Interest Statement

The authors declare that the research was conducted in the absence of any commercial or financial relationships that could be construed as a potential conflict of interest.

The reviewer LC and handling Editor declared their shared affiliation and the handling Editor states that the process nevertheless met the standards of a fair and objective review.

## Footnotes

1 www.ncbi.nlm.nih.gov/blast

## References

[B1] Abd-Alla M. H. El-Sayed E. S. A. Rasmey A. H. M. (2013). Indole-3-acetic acid (IAA) production by *Streptomyces atrovirens* isolated from rhizospheric soil in Egypt. *J. Biol. Earth Sci.* 3 182–193.

[B2] Aghamirian M. R. Ghiasian S. A. (2009). Isolation and characterization of medically important aerobic actinomycetes in soil of iran (2006-2007). *Open Microbiol. J.* 3 53–57. 10.2174/1874285800903010053 19440253 PMC2681176

[B3] Aly A. M. Adel A. El-Gendy A. O. Essam T. M. Aziz R. K. (2016). Gut microbiome alterations in patients with stage 4 hepatitis C. *Gut Pathog.* 8 42 10.1186/s13099-016-0124-2 PMC5020480 27625705

[B4] Atta H. M. (2009). An antifungal agent produced by *Streptomyces olivaceiscleroticus*, AZ-SH514. *World Appl. Sci. J.* 6 1495–1505.

[B5] Awad H. M. El-Sahed K. El-Nakkadi A. (2009). Isolation, screening and identification of newly isolated soil *Streptomyces* (*Streptomyces* sp. NRC-35) for b-Lactamase inhibitor production. *World Appl. Sci. J.* 7 637–646.

[B6] Baker G. Smith J. J. Cowan D. A. (2003). Review and re-analysis of domain-specific 16S primers. *J. Microbiol. Methods* 55 541–555. 10.1016/j.mimet.2003.08.009 14607398

[B7] Birri D. J. Brede D. A. Tessema G. T. Nes I. F. (2013). Bacteriocin production, antibiotic susceptibility and prevalence of haemolytic and gelatinase activity in faecal lactic acid bacteria isolated from healthy Ethiopian infants. *Microb. Ecol.* 65 504–516. 10.1007/s00248-012-0134-7 23184155

[B8] Bizuye A. Moges F. Andualem B. (2013). Isolation and screening of antibiotic producing actinomycetes from soils in Gondar town, North West Ethiopia. *Asian Pac. J. Trop. Dis.* 3 375–381. 10.1016/S2222-1808(13)60087-0

[B9] Busti E. Monciardini P. Cavaletti L. Bamonte R. Lazzarini A. Sosio M. (2006). Antibiotic-producing ability by representatives of a newly discovered lineage of actinomycetes. *Microbiology* 152 675–683. 10.1099/mic.0.28335-0 16514148

[B10] Dilip C. V. Mulaje S. Mohalkar R. (2013). A review on actinomycetes and their biotechnological application. *Int. J. Pharm. Sci. Res.* 4 1730–1742.

[B11] Donadio S. Monciardini P. Sosio M. (2007). Polyketide synthases and nonribosomal peptide synthetases: the emerging view from bacterial genomics. *Nat. Prod. Rep.* 24 1073–1109. 10.1039/b514050c 17898898

[B12] Dundar H. Brede D. A. La Rosa S. L. El-Gendy A. O. Diep D. B. Nes I. F. (2015). The fsr quorum-sensing system and cognate gelatinase orchestrate the expression and processing of proprotein EF_1097 into the Mature antimicrobial peptide enterocin O16. *J. Bacteriol.* 197 2112–2121. 10.1128/JB.02513-14 25733609 PMC4455272

[B13] Einholm A. P. Pedersen K. E. Troels W. Kulig P. Overgaard M. T. Jensen J. K. (2003). Biochemical mechanism of action of a diketopiperazine inactivator of plasminogen activator inhibitor-1. *Biochem. J.* 373 723–732. 10.1042/BJ20021880 12723974 PMC1223537

[B14] El-Hawary S. Mohammed R. AbouZid S. Ali Z. Y. El-Gendy A. O. Elwekeel A. (2016). In-vitro cyclooxygenase Inhibitory, antioxidant and antimicrobial activities of phytochemicals isolated from *Crassula arborescens* (Mill.) Willd. *Int. J. Appl. Res. Nat. Prod.* 9 8–14.

[B15] Fukuchi N. Futaki F. Kito M. Sato S. Kajiura T. Ono Y. (2009). Substance with Antithrombotic Activity and Method for Detecting Glycokallidin. US 7608695.

[B16] Goodfellow M. Williams S. (1983). Ecology of actinomycetes. *Annu. Rev. Microbiol.* 37 189–216. 10.1146/annurev.mi.37.100183.001201 6357051

[B17] Hong-Thao P. T. Mai-Linh N. V. Hong-Lien N. T. Van Hieu N. (2016). Biological characteristics and antimicrobial activity of endophytic *Streptomyces* sp. *TQR*12-4 isolated from Elite Citrus nobilis cultivar ham yen of vietnam. *Int. J. Microbiol.* 2016 1–7. 10.1155/2016/7207818 PMC5067319 27795709

[B18] Hozzein W. N. Goodfellow M. (2007). *Streptomyces synnematoformans* sp. nov., a novel actinomycete isolated from a sand dune soil in Egypt. *Int. J. Syst. Evol. Microbiol.* 57 2009–2013. 10.1099/ijs.0.65037-0 17766864

[B19] Isik K. Gencbay T. Özdemir-Kocak F. Cil E. (2014). Molecular identification of different actinomycetes isolated from East Black Sea region plateau soil by 16S rDNA gene sequencing. *Afr. J. Microbiol. Res.* 8 878–887. 10.5897/AJMR2013.6174

[B20] Jayatilake G. S. Thornton M. P. Leonard A. C. Grimwade J. E. Baker B. J. (1996). Metabolites from an Antarctic sponge-associated bacterium, *Pseudomonas aeruginosa*. *J. Nat. Prod.* 59 293–296. 10.1021/np960095b 8882433

[B21] Kadiri S. K. Yarla N. S. (2016). Optimization of antimicrobial metabolites production by *Streptomyces fradiae*. *Int. J. Pharm. Pharm. Sci.* 7 223–225.

[B22] Kimura M. (1980). A simple method for estimating evolutionary rates of base substitutions through comparative studies of nucleotide sequences. *J. Mol. Evol.* 16 111–120. 10.1007/BF01731581 7463489

[B23] Kumar S. Stecher G. Tamura K. (2016). MEGA7: Molecular Evolutionary Genetics Analysis version 7.0 for bigger datasets. *Mol. Biol. Evol.* 33 1870–1874. 10.1093/molbev/msw054 27004904 PMC8210823

[B24] Li X. Dobretsov S. Xu Y. Xiao X. Hung O. S. Qian P. (2006). Antifouling diketopiperazines produced by a deep-sea bacterium, *Streptomyces fungicidicus*. *Biofouling* 22 187–194. 10.1080/08927010600780771 17290864

[B25] Martins M. B. Carvalho I. (2007). Diketopiperazines: biological activity and synthesis. *Tetrahedron* 63 9923–9932. 10.1016/j.tet.2007.04.105

[B26] Matselyukh B. Mohammadipanah F. Laatsch H. Rohr J. Efremenkova O. Khilya V. (2012). Purification and structure elucidation of the by-product of new regulator of antibiotic production and differentiation of *Streptomyces*. *Mikrobiol. Z.* 74 66–73. 23120988

[B27] Olano C. Méndez C. Salas J. A. (2009). Antitumor compounds from actinomycetes: from gene clusters to new derivatives by combinatorial biosynthesis. *Nat. Prod. Rep.* 26 628–660. 10.1039/b822528a 19387499

[B28] Pandey A. Shukla A. Majumdar S. (2005). Utilization of carbon and nitrogen sources by *Streptomyces kanamyceticus* M 27 for the production of an Anti bacterial antibiotic. *Afr. J. Biotechnol.* 4 909–910.

[B29] Passari A. K. Mishra V. K. Saikia R. Gupta V. K. Singh B. P. (2015). Isolation, abundance and phylogenetic affiliation of endophytic actinomycetes associated with medicinal plants and screening for their in vitro antimicrobial biosynthetic potential. *Front. Microbiol.* 6:273 10.3389/fmicb.2015.00273 PMC4388002 25904906

[B30] Reddy N. Ramakrishna D. Raja Gopal S. (2011). A morphological, physiological and biochemical studies of marine *Streptomyces rochei* (MTCC 10109) showing antagonistic activity against selective human pathogenic microorganisms. *Asian J. Biol. Sci.* 4 1–14. 10.3923/ajbs.2011.1.14

[B31] Rifaat H. M. Abd El Naser N. H. Helmy S. M. Ali A. M. (2013). Taxonomical studies of certain streptomycetes exhibiting antimicrobial activity isolated from Egyptian soils. *J. Cult. Collect.* 5 25–34

[B32] Romankova A. Zurabova E. Fursenko M. Sukharevich V. Pronina M. (1971). Selection of strains of some antibiotic producing Actinomycetes during repeated passages in submerged cultures. *Antibiotiki* 16 579–583. 5168223

[B33] Sacramento D. R. Coelho R. R. R. Wigg M. D. Toledo Luna Linhares L. F. D. Matos dos Santos M. G. Azevedo Soares Semêdo L. T. D. (2004). Antimicrobial and antiviral activities of an actinomycete (*Streptomyces* sp.) isolated from a Brazilian tropical forest soil. *World J. Microbiol. Biotechnol.* 20 225–229. 10.1023/B:WIBI.0000023824.20673.2f

[B34] Saitou N. Nei M. (1987). The neighbor-joining method: a new method for reconstructing phylogenetic trees. *Mol. Biol. Evol.* 4 406–425. 3447015 10.1093/oxfordjournals.molbev.a040454

[B35] Savic M. Bratic I. Vasiljevic B. (2007). *Streptomyces durmitorensis* sp. nov., a producer of an FK506-like immunosuppressant. *Int. J. Syst. Evol. Microbiol.* 57 2119–2124. 10.1099/ijs.0.64913-0 17766883

[B36] Selvameenal L. Radhakrishnan M. Balagurunathan R. (2009). Antibiotic pigment from desert soil actinomycetes; biological activity, purification and chemical screening. *Indian J. Pharm. Sci.* 71 499–504. 10.4103/0250-474X.58174 20502566 PMC2866339

[B37] Selvin J. Shanmughapriya S. Gandhimathi R. Kiran G. S. Ravji T. R. Natarajaseenivasan K. (2009). Optimization and production of novel antimicrobial agents from sponge associated marine actinomycetes *Nocardiopsis dassonvillei* MAD08. *Appl. Microbiol. Biotechnol.* 83 435–445. 10.1007/s00253-009-1878-y 19190903

[B38] Ser H. L. Palanisamy U. D. Yin W. F. Chan K. G. Goh B. H. Lee L. H. (2016a). *Streptomyces malaysiense* sp. nov.: a novel Malaysian mangrove soil actinobacterium with antioxidative activity and cytotoxic potential against human cancer cell lines. *Sci. Rep.* 6:24247 10.1038/srep24247 PMC4829849 27072394

[B39] Ser H. L. Palanisamy U. D. Yin W. F. Malek S. N. A. Chan K. G. Goh B. H. (2015). Presence of antioxidative agent, Pyrrolo [1, 2-a] pyrazine-1, 4-dione, hexahydro-in newly isolated *Streptomyces mangrovisoli* sp. *nov.* *Front. Microbiol.* 6:854 10.3389/fmicb.2015.00854 PMC4542459 26347733

[B40] Ser H. L. Tan L. T. H. Palanisamy U. D. Malek S. N. A. Yin W. F. Chan K. G. (2016b). *Streptomyces* antioxidans sp. nov., a novel mangrove soil actinobacterium with antioxidative and neuroprotective potentials. *Front. Microbiol.* 7:899 10.3389/fmicb.2016.00899 PMC4909769 27379040

[B41] Sharma P. Kalita M. C. Thakur D. (2016). Broad spectrum antimicrobial activity of forest-derived soil actinomycete, *Nocardia* sp. PB-52. *Front. Microbiol.* 7:347 10.3389/fmicb.2016.00347 PMC4796592 27047463

[B42] Sinha S. Srivastava R. De Clercq E. Singh R. K. (2004). Synthesis and antiviral properties of arabino and ribonucleosides of 13-dideazaadenine, 4-nitro-1, 3-dideazaadenine and diketopiperazine. *Nucleosides Nucleotides Nucleic Acids* 23 1815–1824. 10.1081/NCN-200040614 15628741

[B43] Skehan P. Storeng R. Scudiero D. Monks A. McMahon J. Vistica D. (1990). New colorimetric cytotoxicity assay for anticancer-drug screening. *J. Natl. Cancer Inst.* 82 1107–1112. 10.1093/jnci/82.13.1107 2359136

[B44] Stackebrandt E. Charfreitag O. (1990). Partial 16S rRNA primary structure of five *Actinomyces* species: phylogenetic implications and development of an *Actinomyces israelii*-specific oligonucleotide probe. *Microbiology* 136 37–43. 10.1099/00221287-136-1-37 1693659

[B45] Sujatha P. Raju K. B. Ramana T. (2005). Studies on a new marine streptomycete BT-408 producing polyketide antibiotic SBR-22 effective against methicillin resistant *Staphylococcus aureus*. *Microbiol. Res.* 160 119–126. 10.1016/j.micres.2004.10.006 15881828

[B46] Tamura K. Dudley J. Nei M. Kumar S. (2007). MEGA4: molecular evolutionary genetics analysis (MEGA) software version 4.0. *Mol. Biol. Evol.* 24 1596–1599. 10.1093/molbev/msm092 17488738

[B47] Tan L. Ser H. Yin W. Chan K. Lee L. Goh B. (2015). Investigation of antioxidative and anticancer potentials of *Streptomyces* sp. MUM256 isolated from Malaysia Mangrove Soil. *Front. Microbiol.* 6:1316 10.3389/fmicb.2015.01316 PMC4659911 26635777

[B48] Thakur D. Yadav A. Gogoi B. Bora T. (2007). Isolation and screening of *Streptomyces* in soil of protected forest areas from the states of Assam and Tripura, India, for antimicrobial metabolites. *J. Mycol. Med.* 17 242–249. 10.1016/j.mycmed.2007.08.001

[B49] Undabarrena A. Beltrametti F. Claverías F. P. González M. Moore E. R. Seeger M. (2016). Exploring the diversity and antimicrobial potential of marine actinobacteria from the Comau Fjord in Northern Patagonia, Chile. *Front. Microbiol.* 7:1135 10.3389/fmicb.2016.01135 PMC4949237 27486455

[B50] Wang P. Xi L. Liu P. Wang Y. Wang W. Huang Y. (2013). Diketopiperazine derivatives from the marine-derived actinomycete *Streptomyces* sp. FXJ7. 328. *Mar. Drugs* 11 1035–1049. 10.3390/md11041035 23538868 PMC3705386

[B51] Williams S. Davies F. (1965). Use of antibiotics for selective isolation and enumeration of actinomycetes in soil. *Microbiology* 38 251–261. 10.1099/00221287-38-2-251 14287203

[B52] Williams S. Goodfellow M. Alderson G. Wellington E. Sneath P. Sackin M. (1983). Numerical classification of *Streptomyces* and related genera. *Microbiology* 129 1743–1813. 10.1099/00221287-129-6-1743 6631406

[B53] Wood S. Kirby B. Goodwin C. Le Roes M. Meyers P. (2007). PCR screening reveals unexpected antibiotic biosynthetic potential in *Amycolatopsis* sp. strain UM16. *J. Appl. Microbiol.* 102 245–253. 10.1111/j.1365-2672.2006.03043.x 17184341

