# Supplementary material for: Exploring the Antimicrobial and Antitumor Potentials of Streptomyces sp. AGM12-1 Isolated from Egyptian Soil
Source: Front Microbiol. 2017 Mar 13;8:438. doi: 10.3389/fmicb.2017.00438 (PMC5346535; doi:10.3389/fmicb.2017.00438)
Supplement: Supplementary file 1 [file Table_1.DOCX]

**Supplementary table 1a:** screening of antimicrobial activity of 20 Actinomycete isolates against different indicator microorganisms by double layer agar method (modified spot on lawn technique)**.**

| **Isolate code number** | **Mean values of inhibition zones of growth(mm) against:** | | | | |
| --- | --- | --- | --- | --- | --- |
|  | *S. lutea* | *C. albicans* | *St. aureus* | *E. coli* | *B. subtilis* |
| **AGM1-2** | 46 | 23 | 12 | 25 | 41 |
| **AGM1-5** | 32 | 0 | 0 | 0 | 0 |
| **AGM2-1** | 25 | 0 | 24 | 22 | 34 |
| **AGM3-1** | 0 | 0 | 0 | 40 | 38 |
| **AGM5-1** | 18 | 22 | 0 | 21 | 0 |
| **AGM8-1** | 16 | 0 | 0 | 0 | 0 |
| **AGM8-2** | 22 | 0 | 0 | 0 | 0 |
| **AGM8-3** | 27 | 0 | 0 | 0 | 0 |
| **AGM8-4** | 16 | 0 | 0 | 0 | 0 |
| **AGM8-8** | 14 | 0 | 0 | 0 | 0 |
| **AGM8-9** | 16 | 0 | 0 | 0 | 0 |
| **AGM10-1** | 32 | 0 | 0 | 0 | 0 |
| **AGM10-3** | 11 | 0 | 0 | 0 | 0 |
| **AGM11-4** | 14 | 0 | 0 | 0 | 0 |
| **AGM11-5** | 20 | 0 | 28 | 0 | 0 |
| **AGM12-1** | 44 | 28 | 16 | 20 | 26 |
| **AGM12-3** | 34 | 16 | 19 | 16 | 16 |
| **AGM13-1** | 40 | 30 | 17 | 17 | 28 |
| **AGM13-4** | 19 | 0 | 0 | 0 | 0 |
| **AGM13-10** | 34 | 15 | 0 | 22 | 34 |

**Supplementary table 1b:** Screening of antimicrobial activity of 20 Actinomycete isolates against different indicator microorganisms by cup method assay.

| **Isolate code number** | **Mean values of inhibition zones of growth(mm) against:** | | | | |
| --- | --- | --- | --- | --- | --- |
|  | *S.lutea* | *C.albicans* | *St. aureus* | *E, coli* | *B.subtilis* |
| **AGM1-2** | 16 | 15 | 0 | 19 | 20 |
| **AGM1-5** | 18 | 0 | 0 | 0 | 0 |
| **AGM2-1** | 21 | 0 | 22 | 22 | 17 |
| **AGM3-1** | 0 | 0 | 0 | 22 | 14 |
| **AGM5-1** | 15 | 19 | 0 | 18 | 0 |
| **AGM8-1** | 12 | 0 | 0 | 0 | 0 |
| **AGM8-2** | 18 | 0 | 0 | 0 | 0 |
| **AGM8-3** | 20 | 0 | 0 | 0 | 0 |
| **AGM8-4** | 12 | 0 | 0 | 0 | 0 |
| **AGM8-8** | 13 | 0 | 0 | 0 | 0 |
| **AGM8-9** | 14 | 0 | 0 | 0 | 0 |
| **AGM10-1** | 19 | 0 | 0 | 0 | 0 |
| **AGM10-3** | 11 | 0 | 0 | 0 | 0 |
| **AGM11-4** | 17 | 0 | 0 | 0 | 0 |
| **AGM11-5** | 14 | 0 | 19 | 0 | 0 |
| **AGM12-1** | 19 | 16 | 13 | 14 | 16 |
| **AGM12-3** | 17 | 11 | 13 | 15 | 14 |
| **AGM13-1** | 20 | 15 | 12 | 12 | 16 |
| **AGM13-4** | 16 | 0 | 0 | 0 | 0 |
| **AGM13-10** | 20 | 11 | 0 | 14 | 19 |
