# Supplementary material for: Exploring the Antimicrobial and Antitumor Potentials of Streptomyces sp. AGM12-1 Isolated from Egyptian Soil
Source: Front Microbiol. 2017 Mar 13;8:438. doi: 10.3389/fmicb.2017.00438 (PMC5346535; doi:10.3389/fmicb.2017.00438)
Supplement: Supplementary file 2 [file Image_1.PDF]

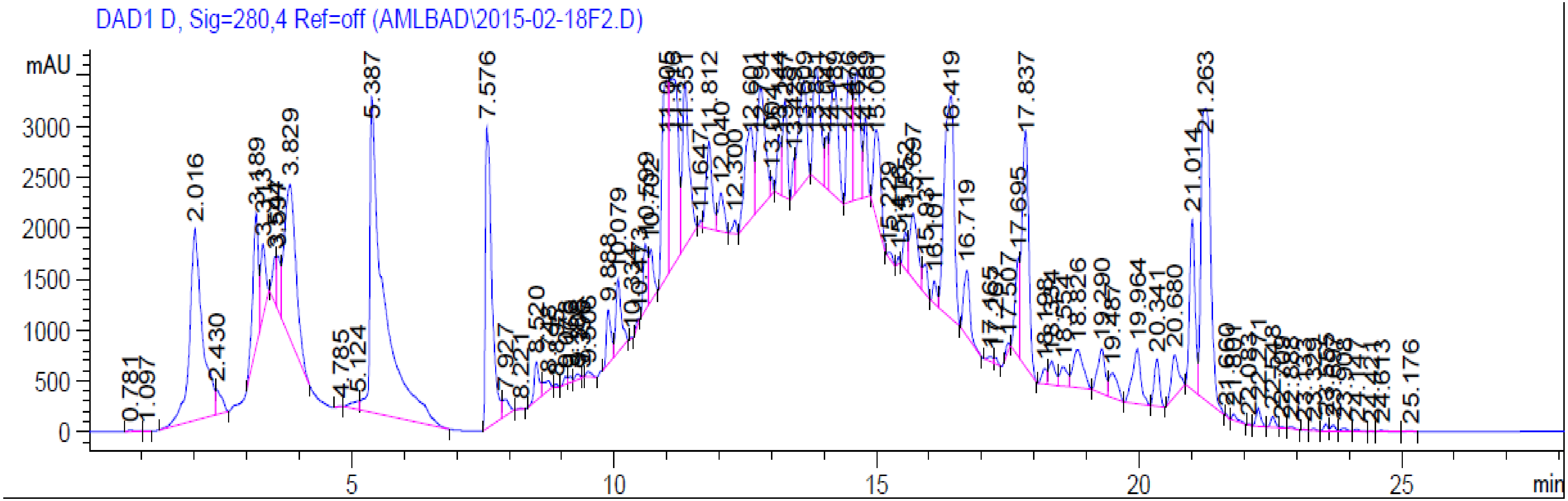

Supplementary Figure 1: First run of HPLC using a Nucleosil C18 column showing multiple peaks and compounds in ethyl acetate extract of AGM12-1 isolate.
